# Clinical Lecture on Paralysis

**Published:** 1857-05

**Authors:** Samuel Solly

**Affiliations:** Surgeon to St. Thomas’s Hospital


*Clinical Lecture on Paralysis.* By Samuel Solly, Esq., Surgeon to St. Thomas's Hospital.

LECTURE I.

*Gentlemen.*—Two cases of paraplegia, or palsy of the lower half of the body, now under my care in this hospital, enable me to call your attention in this course of Clinical Lectures to the important subject of paralysis. Paralysis is a term much used out of the profession, and but little understood. It is employed by non-professional persons to designate a cause, not an effect. It is therefore possible that many of you, who are now only commencing your studies, still in the embryonic condition of medical pupilage, may have the same vague ideas of the meaning of the term, and none, I am sure, can know too much, think too deeply, or observe too closely, all that relates to the important subject. The practitioner who can diagnose correctly the causes of paralysis in its early states, will often save both the life and the intellect of his patient; the man who mistakes it, often sacrifices both to his ignorance. The diagnosis and treatment of paralysis fall alike to the physician, the surgeon, and the general practitioner. Woe to you, my young friends, if you do not avail yourselves of the opportunities which the large wards of this noble hospital afford. In a clinical lecture, I shall not of course, enter into a minute disquisition on paralysis, but I must say a few words for the instruction of my younger hearers.

Paralysis may be general or local. Its proximate or immediate cause may be pressure on some portion of the nervous system, or disorganisation of it. The ultimate cause may be local violence, such as a fracture of the skull or the vertebral column, effusion of blood as in apoplexy, or a mere "coup de vent," or blast of cold air on the face, inducing facial paralysis or palsy of the portio dura. I have lately had two well-marked cases of local paralysis from a railway accident. In the one there was a deep lacerated wound above the eye, which divided the supra-orbital nerve, and the upper part of the forehead was quite numb; there was entire loss of sensation in that portion of the skin which is supplied in a normal state by that nerve. In the other case the auricular nerve, one of the sensory branches of the fifth, was torn through under the skin without any external wound; the skin covering that side of the head was devoid of sensation.

That form of blindness which the ophthalmic surgeon knows under the title of amaurosis, and the public by the name of gutta serena, is a palsy of the optic nerve, sometimes induced by pressure in the globe of the eye, sometimes by pressure on the nerve in its course from the brain, and sometimes by disease of the brain itself; and so I might continue for the next half hour to give you illustrations of individual forms of paralysis; but trusting that I have said enough to make you understand the meaning of the term, I shall advance at once to special instances of this disease, in the hope of assisting you to distinguish some of its most important and most frequently recurring forms. I wish to assist you especially to distinguish between paralysis arising from disease or injury of the spinal cord and that from disease or injury of the brain. Now, many of the older students will perhaps think this a most easy matter —that a man must be a fool who cannot do so at once—but I assure you that this is a mistake.

In the early stages of paralysis it is often by no means easy to do so. I have lately seen two cases in private practice, in which it was difficult to diagnose the seat of the disease. In the one, a case of spinal paralysis, the disease presented so many of the characters of the general palsy of the insane, that a very intelligent practitioner was inclined to regard it as one of the instances of that sad and I believe irremediable disease. The other, which has since proved to be a complete case of cerebral palsy, was in its early stages supposed to be a true spinal affection. In the first case the patient is recovering; in the other he is sinking into a state of hopeless dementia. As I shall relate the first case at length, I will not say more about it at present. Of the case of general paralysis and dementia I will say a few words.

The subject of it is a man who was once as strong and as healthy as any one of you, but his business was an exciting one, requiring great energy, and tasking the brain to its utmost. In order to supply, and, as he believed, by necessity, the waste which his mental and bodily work created, he used to take a large quantity of wine, thus adding fuel to the fire which was kindled within him. I do not mean that he was intemperate in a worldly sense, for a man may take a great deal more of stimulants than is beneficial to his organization without exhibiting any signs of injury at the time; but of this be certain, that if you want to keep your brains in a state of healthful mental activity, you will take very little. The country gentleman and farmer of the old school might drink their wine, their brandy, and their beer with comparative impunity, for their brains were dormant, and these stimulants were the only stimulants their brains received; but woe to the man of intellect, the man who has to live by the sweat of his brain, if he attempts to supply by fermented liquors the loss occasioned by mental labour. He may feel better for a time, but he is sure to sink more rapidly in the end. There was another habit, also, in which my patient indulged, and which I cannot but regard as the curse of the present age. I mean smoking. Now, don't be frightened, my young friends, I am not going to give a sermon against smoking, that is not my business; but it is my business to point out to you all the various and insidious causes of general paralysis, and smoking is one of them. I know of no *single* vice which does so much harm as smoking. It is a snare and a delusion. It soothes the excited nervous system at the time, to render it more irritable and more feeble ultimately. It is like opium in that respect, and if you want to know all the wretchedness which this drug can produce, you should read the "Confessions of an Opium-eater." I can always distinguish by his complexion a man who smokes much, and the appearance which the fauces present is an unerring guide to the habits of such a man.

I believe that cases of general paralysis are more frequent in England than they used to be, and I suspect that smoking tobacco is one of the causes of that increase.

But I must not detain you any longer from the immediate subject of this clinique. The two cases now in the hospital that I am about to relate, from the notes of my dresser, Mr. Sprakeling, are both cases of spinal paralysis, the one induced by the pressure of an angular curvature of the dorsal portion of the vertebral canal, the other by a blow on the lumbar portion.

William W———, aged thirty-two, compositor, was admitted into Abraham's ward on the 17th of June, 1856. He is an unhealthy, strumous looking man, who states that he never noticed any projection or curvature of the spine till six months ago, but since that time has noticed it gradually coming on. (Let me here remark that this angular curvature is almost always a strumous disease, commencing in the cancellated structure of the bodies of the vertebræ. If you look at this preparation, you will see exactly how it occurs. The body of one or more of the vertebræ being absorbed, the bones above and below fall forward, so as to meet and supply the vacancy. If it were not for this arrangement, our patient's life would not be worth an hour's purchase; for the beautiful protective apparatus of the spinal cord being deficient, its delicate and soft substance would be torn in the first movement that was made. Instead of being slightly pressed, as at present, it would be divided. The angle of the back is the proof that the column is not separated in front.) About six weeks ago, he first began to be sensible of some alteration of temperature in the lower limbs, with numbness and occasional twitchings and rigidity of them. He then began to lose power in them, and for the last three weeks they have been totally paralyzed. At present, there appears to be an angular curvature of the spine in the dorsal region; he seems to have lost the use of the lower extremities entirely, but, with the exception of the feet there is no very perceptible coldness; he has, however, lost almost entirely the sensibility of them. There are occasional spasmodic twitchings and startings of the limbs, but there does not appear to be any tightness over the chest, or dyspnœa. The bowels are costive, but he has not lost control of the sphincters. He has, at times, some difficulty in micturating, with frequent desire to do so, but inability properly to empty his bladder. There appears at present considerable tympanitis, but no great distension of the bladder. His appetite is deficient; urine clear and unsedimentous; pulse 92, of considerable power; tongue clean. Ordered mercury with chalk, two grains every night. A moxa on each side of the spine.

(Believing that the cause of the paralysis in this case is the pressure caused by affusion into the canal at the seat of the angular curvature, I have ordered those remedies which I think are more likely to promote the absorption of the offending matter.) He has never injured the spine from a blow or a fall.

*June* 25. States that he has felt some tingling in the toes and foot, but there is no increase of sensibility in the paralyzed limbs. He is suffering from indigestion. Dyspeptic mixture, one ounce, to be taken twice a day.

27*th*. There seems to be a slight increase of sensibility in the left foot and leg. He suffers a good deal from tympanitis. The bowels are only relieved by aperients.

*July* 5. There is still a good deal of tympanitis, and he complains, and has complained for this last week, of pain in the right hypochondriac region, where there is some tenderness on pressure. The bowels are relaxed. There is a decided increase of sensation in both legs. Pulse small and feeble; tongue clean. Aspect rather improved, as also in his appetite.

11*th*. Complained on the 9th of a good deal of pain in the bowels and in the right hypochondrium. Ordered, iodide of mercury, half a grain, every night. To-day he seems somewhat relieved from the pain, but complains, of a good deal of general weakness. Pulse 84, weak; tongue clean.

19*th*. He does not complain of so much pain in the right hypochondrium or in the bowels. There has been no further improvement in sensation; there are dull aching pains now and then in the legs, with spasmodic startings of them. (I regard these aching pains as a favorable sign; they always precede the natural sensation in the part. I dare say that some of you who are working hard at your profession all day in the hospital have a nap afterwards, previous to commencing your evening work. Occasionally, one of your legs falls asleep, as the ordinary expression is, and it does not awake with the rest of the body. Your leg, in fact, is numb and powerless from pressure on the nerves, usually the popliteal. Now, you must all have remarked that before the natural sensation returns, a most unnatural and painful sensation precedes it—a tingling, or "pins and needles," as we call it. This, on a small scale, and acting very quickly in your persons, is identical with that which is going on more slowly, but I believe as surely, in this patient.) The tongue is clean. The moxa having healed, a fresh one was made to-day.

26*th*. He continues much the same.

31*st*. Much in the same state.

*Aug*. 3. The moxa repeated. Ordered mixed diet.

21*st*. He has improved but slightly. He states that he often feels pricking sensations and startings in the limbs. Sensation has slightly increased, but there is no power of locomotion. His bowels have lately been much confined, but he has experienced considerable relief, and has felt himself better, after an aperient taken yesterday. He states also that his appetite has much improved, and he feels altogether stronger since he has taken the cod-liver oil. Pulse 84, firmer.

26*th*. He thinks that sensation in his legs has further increased. Ordered, mercury with chalk, two grains every night.

*Sept*. 20. Repeat moxa. Compound rhubarb pill, five grains every night.

*Oct*. 8. Within this last fortnight he fancies that there has been a diminution of sensation. Up to this time the paralytic symptoms have remained much in the same state. At present, he has very little feeling in the right leg when touched, sensation appearing to be more perfect in the left. His general health, up to the last three or four days, has improved; he is now, however, complaining of being weak and low-spirited; pulse 76, small and weak; bowels are now confined. To take, iodide of mercury, half a grain, every night.

14*th*. The paralytic symptoms remain much in the same state. Sensation varies a good deal, being more perfect on one day than on another, but the power of locomotion has not increased. He is often troubled to retain his urine. The state of the bowels varies, being sometimes relaxed, and sometimes confined; pulse small and weak.

*Nov*. 7. Still improving.

I have very great hopes that this man will perfectly recover. It may require some faith on your part to believe me when I say that those limbs which are now so senseless and motionless will again support his body, obey his commands, and be recognized again by their sensation as a part of his living structure. I have seen and published the recovery of cases quite as unpromising as this. It may take some months yet to accomplish it, but happily, in a hospital, we are not liable to be cut off in our course of treatment by the impatience of the patient or his friends.

In the next case we shall find the improvement more rapid.

Eleanor V———, aged fifty, housekeeper, admitted into Queen's Ward, August 19th, 1856. She is a hearty, strong woman, of florid complexion, who states that she has occasionally suffered from rheumatism, and had an attack of fever twelve years ago, but, with these exceptions, had always enjoyed good health up to her present illness. She states that her mother and three sisters died consumptive, but her father was always a healthy man, and died at the age of seventy-three years. She ascribes her present condition to a fall she had down-stairs two years ago, whereby she hurt "the lower part of her part." Directly after this fall she felt total loss of power in the legs, with numbness, which lasted about an hour, after which she was able to get up and walk about. Soon afterwards, however, she noticed great coldness of the lower extremities, with loss of power in them, which symptoms have gradually increased up to the present time. There is now some coldness of the feet, but not of the legs. There is great loss of power in the legs, and a considerable loss of sensation, but no numbness. She can walk, but is obliged to be supported to prevent her falling. She suffers also from startings and prickings in the legs, and when she moves them, she states that she feels pain in the back. There is no abnornal curvature or malformation of the spine, but she experiences considerable pain when the second or third lower lumbar vertebræ are struck. There is slight incontinence of urine, but her bowels are regular. Her general health and assimilative powers are good. Pulse 120; tongue slightly coated; she has occasional rigors. There is a small ulcer on the left leg, about the size of a sixpence, with a broad red circumference. She was ordered to take two grains of calomel and half a grain of opium every night. Moxa to be made on the side of the spine; water dressing to be applied to the ulcer.

*Aug.* 27. She thinks that she can move her legs better, and sensation in them has increased; her gums are sore from the mercury. Omit pill.

*Sept.* 3. She has been gradually improving. She can now stand up for a considerable time, and without pain. The issue continues to discharge well. She says she feels herself getting stronger, and can walk from one end of the ward to the other without support. Bowels regular; tongue clean; pulse 98, tolerably firm; appetite good.

11*th*. Still improving. The sensation in her legs has returned perfectly within the last three days, and she can stand up for a longer time than she could. Appetite good; bowels regular.

13*th*. She says that she can feel a sensible improvement in herself every day. Yesterday she could stand up for a much longer time than usual. She is very comfortable and cheerful.

19*th*. Improving daily. Yesterday she was able to walk to the end of the ward and back again without any assistance. Feels very well, but rather weak.

24*th* (Wednesday). Last Saturday she began to sit up all day, and has continued to do so till the present time; but she is not so well to-day, and is weaker in the limbs. She was ordered to keep her bed again, to have another issue made in her back, and to take an ounce and a half of iodine mixture twice a day.

30*th*. Much the same. She has not been out of bed since the issue was made, and feels out of health from having caught cold. Tongue rather furred; bowels regular. Ordered one grain of iodide of mercury and half a grain of opium every night.

*Oct.* 7. She has recovered from the cold, and feels considerably better. Tongue cleaner.

13*th*. The gums are now swollen and tender. She can now raise her legs up in bed, whilst lying in the recumbent position, which she was unable to do on her admission. She also, this morning, walked across the ward without assistance. She is now able to retain the urine, and the bowels act regularly. Sensation in the legs is perfect. She is not troubled with prickings or startings in the legs now. Pulse full; appetite good.

One peculiarity in this case—and it is a peculiarity of great importance in a practical point of view—is the length of time which elapsed between the occurrence of the injury and the paralytic symptoms—nearly two years. Let this fact warn you, when you are engaged in private practice, to give a very guarded prognosis of the consequences which may ensue from a blow on the spine, and let it remind you to inquire particularly as to the antecedents in a case of paraplegia, where the causes are obscure and the diagnosis consequently difficult. This again brings to my mind the case that I referred to at the commencement of the lecture, and which I wish to relate to you in connection with this subject; but I find by the time which has elapsed I must reserve it for another time.—*Lancet*, Dec. 13, 1856.

